# Tropicalization of the barrier islands of the northern Gulf of Mexico: A comparison of herbivory and decomposition rates between smooth cordgrass (*Spartina alterniflora*) and black mangrove (*Avicennia germinans*)

**DOI:** 10.1371/journal.pone.0210144

**Published:** 2019-01-07

**Authors:** Aaron Macy, Shailesh Sharma, Eric Sparks, Josh Goff, Kenneth L. Heck, Matthew W. Johnson, Patric Harper, Just Cebrian

**Affiliations:** 1 Dauphin Island Sea Lab, Dauphin Island, AL, United States of America; 2 University of South Alabama, Marine Science Department, Mobile, AL, United States of America; 3 Mississippi State University Coastal Research and Extension Center, Biloxi, MS, United States of America; 4 Mississippi-Alabama Sea Grant Consortium, Ocean Springs, MS, United States of America; 5 National Marine Fisheries Service, Southeast Fisheries Science Center, Miami, FL, United States of America; 6 Northern Gulf Coastal Program, US Fish and Wildlife Service, Grand Bay Coastal Resources Center, Moss Point, MS, United States of America; Fred Hutchinson Cancer Research Center, UNITED STATES

## Abstract

The expansion of black mangrove *Avicennia germinans* into historically smooth cordgrass *Spartina alterniflora*-dominated marshes with warming temperatures heralds the migration of the marsh-mangrove ecotone northward in the northern Gulf of Mexico. With this shift, *A*. *germinans* is expected to outcompete *S*. *alterniflora* where it is able to establish, offering another prevalent food source to first order consumers. In this study, we find *A*. *germinans* leaves to be preferable to chewing herbivores, but simultaneously, chewing herbivores cause more damage to *S*. *alterniflora* leaves. Despite higher nitrogen content, *A*. *germinans* leaves decomposed slower than *S*. *alterniflora* leaves, perhaps due to other leaf constituents or a different microbial community. Other studies have found the opposite in decomposition rates of the two species’ leaf tissue. This study provides insights into basic trophic process, herbivory and decomposition, at the initial stages of black mangrove colonization into *S*. *alterniflora* salt marsh.

## Introduction

As climates warm, tropical species are expanding their population ranges into historically subtropical environments [1], and lack of severe freeze events as the climate warms is expected to allow for more permanent expansion of mangrove populations in the northern Gulf of Mexico (nGOM) [2] and around the world [3, 4], rather than the alternating expansion/contraction cycles witnessed in the past. Coastal wetlands of the nGOM are largely dominated by herbaceous marsh comprised of smooth cordgrass *Spartina alterniflora* and/or black needlerush *Juncus roemerianus*, though some small regions of warmer climate within the nGOM contain stunted populations of the black mangrove *Avicennia germinans*. The Intergovernmental Panel on Climate Change predicts fewer severe cold events in the coming decades [5], which would facilitate continued poleward expansion of *A*. *germinans* and a gradual conversion of coastal wetlands along the nGOM from herbaceous marsh to woody mangrove swamps [2].

Along with temperature, other factors also limit the establishment and growth of *A*. *germinans*, such as wave action [6], propagule dispersal [7], propagule viability [8], microclimate [9], and hydrology [10, 11]. *A*. *germinans* often colonizes shorelines populated with *S*. *alterniflora* through herbaceous marsh entrapment of mangrove propagules [7], and this mangrove colonization comes at the expense of some salt marsh [12]. This shift from salt marsh to mangrove is associated with substantial shifts in ecosystems structure and function [13, 14]. This mangrove succession over salt marsh may lead to shifts in energy transfer through consumer preference and detrital pathways. Despite the significance of energy transfer on ecosystems, it can be difficult to quantify in the field.

Studies of herbivory and decomposition rates on mangroves are limited to areas where mangroves are dominant, but these rates likely differ near the edges of their geographic extent due to colder climates and different faunal and microbial assemblages. Duarte & Cebrian [15] conducted an extensive review of carbon budgets in coastal systems, and concluded herbivory amounted to 7–11% of carbon transfer in mangroves and 24–38% carbon transfer in marshes, though more recent work suggests herbivory on mangroves has been underestimated [16, 17]. Decomposition rates vary with environmental conditions, including temperature [18], decomposer community [19], redox potential [20], and nutrient availability [21]. No study to the authors’ knowledge has compared herbivory and decomposition rates of marsh and mangrove at the poleward extent of *A*. *germinans*.

In this study, we focus on a barrier island located at the most northern extent of *A*. *germinans* occurrence in the nGOM [22]. Three *A*. *germinans* individuals were discovered in 2012 to have colonized Horn Island. Though the exact time of colonization is unknown, the trees were 0.72–1.08m tall in April 2012. We carried out a two-year study comparing leaf herbivory, leaf nutrient content, and decomposition rates between *S*. *alterniflora* and *A*. *germinans* at this early stage of *A*. *germinans* colonization. Our results demonstrate a dichotomy in the levels of leaf herbivory, based upon mass or areal removal analysis, and offer novel findings on differences in decomposition rates between *A*. *germinans* and *S*. *alterniflora*, illustrating important consideration of region-specificity in generalizations concerning expanding or migrating species ranges.

## Methods

### Study site

**Fig 1:**
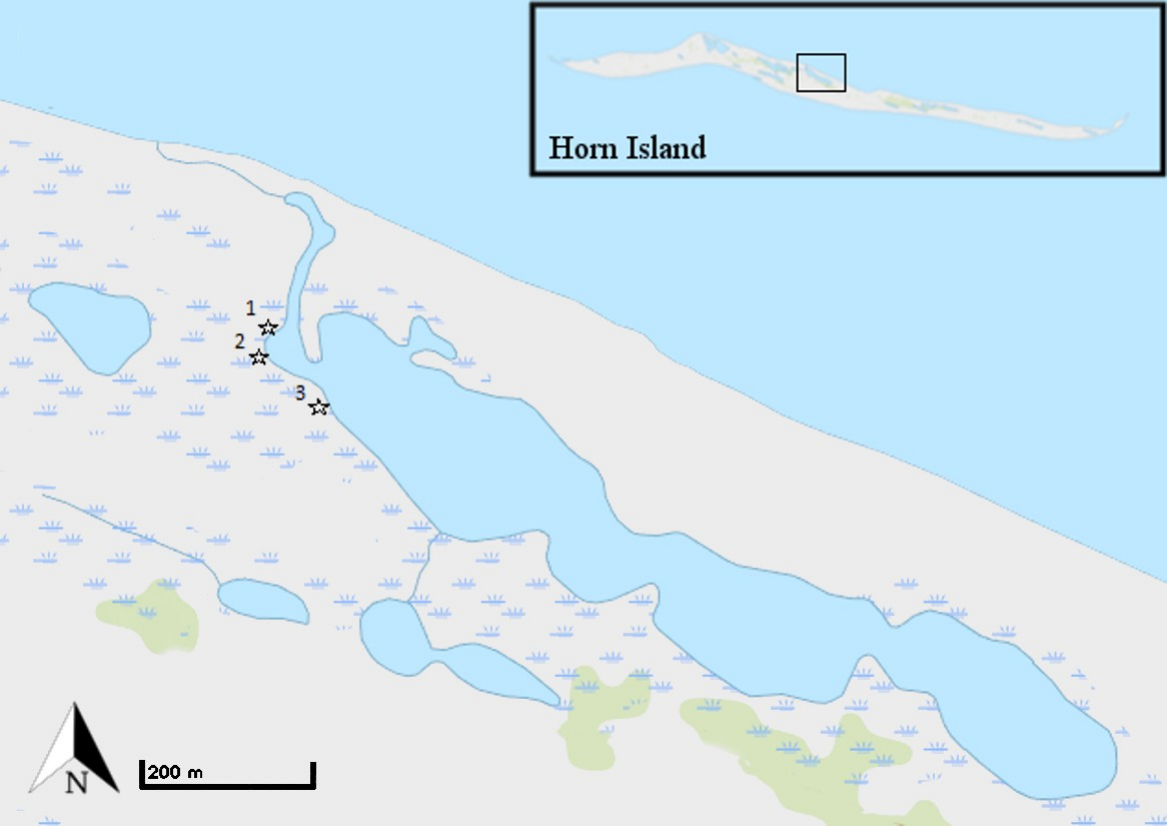
Map of stations 1–3 (stars) on Horn Island, MS. Study region indicated on inset of Horn Island in top right. Each station contained a mixed and reference plot, 5m apart. Mixed (2012–2013) and reference (2013) plot sizes were scaled to the size of the *A*. *germinans* tree in the corresponding station (Stations 1 and 3: 1.0m^2^; Station 2: 0.25m^2^). Imagery of the study site and the Horn Island insert were obtained from USGS National Map Viewer (public domain).

Horn Island is a barrier island off the coast of Mississippi (30°14'27" N, 88°40'43"W) that marks the northern limit of *A*. *germinans* in the Gulf of Mexico [22]. The entire known mangrove population on the island, composed of three individuals at the time of this study (2012–2013), resided along the shoreline of a bayside inlet to a lagoon on the interior of Horn Island (Fig 1). The shoreline was dominated by *S*. *alterniflora* interspersed with patches of black needlerush *J*. *romerianus* and other salt marsh species.

### Study design

For the first year of the study (2012), we established three stations, each comprised of a single “mixed” plot centered on one of the three surveyed *A*. *germinans* trees, thus containing the tree and underlying marsh. Each station was expanded in the second year (2013) to include a second, marsh-only (“reference”), plot, 5m away from its paired mixed plot. *S*. *alterniflora* was the dominant species of marsh plant and referred to as “*S*. *alterniflora*-mixed” in mixed plots and “*S*. *alterniflora*-ref” in reference plots. Mixed and reference plot sizes were scaled to the size of the *A*. *germinans* tree (0.25–1.0m^2^) in the corresponding station (Fig 1).

Floral communities, *A*. *germinans* and *S*. *alterniflora* morphometrics, and herbivory were measured bimonthly over the 2012 and 2013 growing seasons (June-October). Leaf decomposition rates using the conventional litterbag method [23, 24] were measured in 2012; in 2013, many of the litterbags were washed away, precluding analysis of 2013 data, and leaf carbon and nitrogen content were measured bimonthly in 2013.

### Measurements

#### Leaf herbivory

With the permission of the United States Fish and Wildlife Service, thirty green *A*. *germinans* leaves were collected haphazardly from each tree on each sampling date. Thirty live shoots of *S*. *alterniflora* were collected haphazardly from just outside each plot around or adjacent to the tree. Photos were taken of all *A*. *germinans* leaves. *S*. *alterniflora* leaves were peeled off the shoot and their basal terminus defined by a distinct line where the leaf diverges from the stem. Height and basal width were measured for all *S*. *alterniflora* leaves. Photos were taken of the leaf sections with bite marks. *A*. *germinans* leaf area, and the area of the bite marks on *A*. *germinans* and *S*. *alterniflora* leaves were measured with ImageJ software.

To estimate leaf area for *S*. *alterniflora* leaves with tip present, we derived an allometric equation with fifteen *S*. *alterniflora* shoots collected from a marsh on Dauphin Island (30°15'3.56"N, 88° 4'39.91"W). Many *S*. *alterniflora* leaves were missing the tip, particularly those with many bite marks. This was not the case for *A*. *germinans* leaves since grazed leaves maintained their tip and oval shape despite having grazing marks. Missing tips could have endured substantial grazing; thus, disregard of these tips could lead to severe grazing underestimates [25]. We used a two-step approach to derive a possible range of herbivory intensity for leaves where the tip was missing.

**Fig 2:**
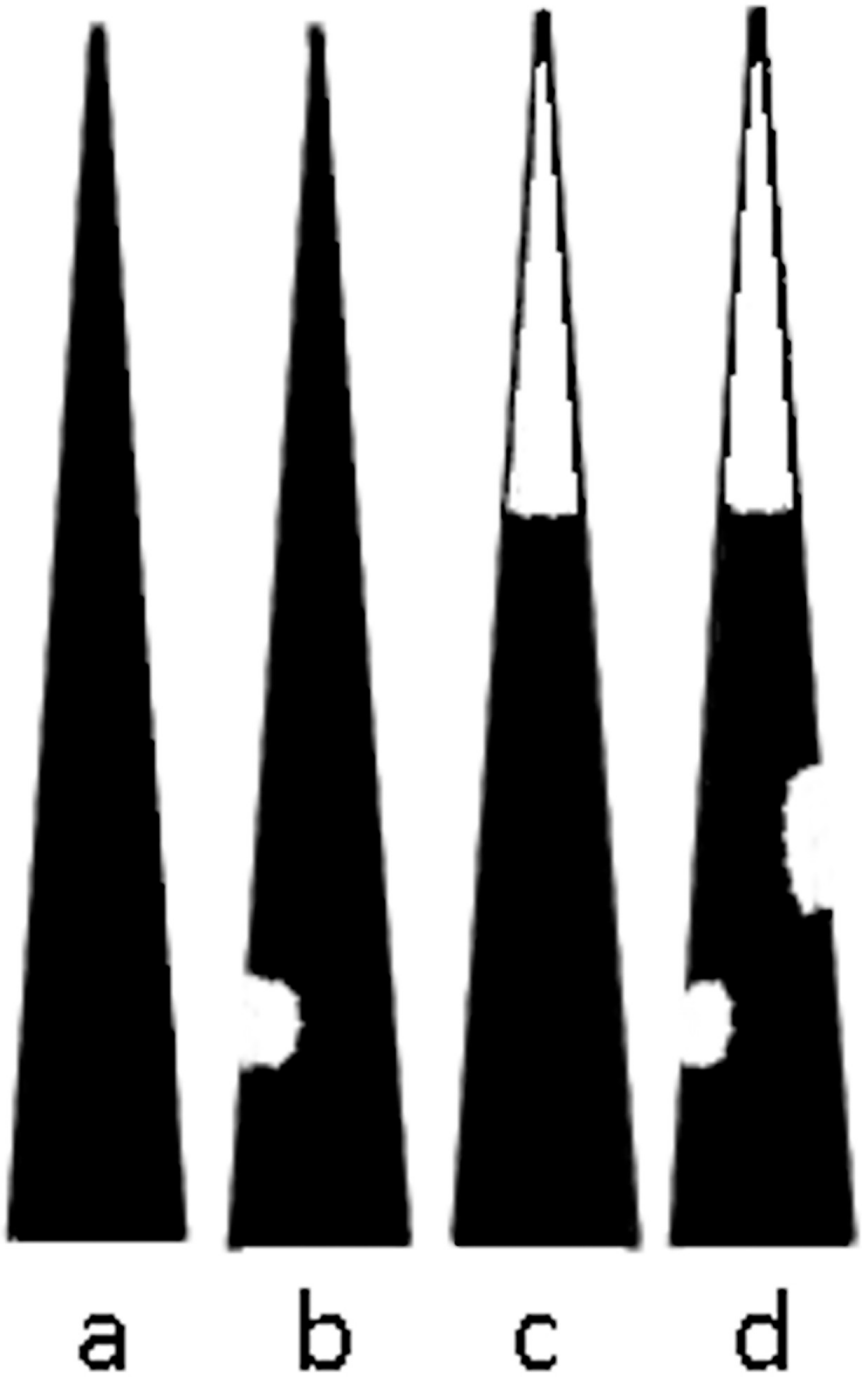
**Four possible situations encountered for *S*. *alterniflora* leaves:** a) leaf tip intact, no grazing (76.1% of leaves), b) leaf tip intact, grazed (6.4% of leaves), c) leaf tip missing, no grazing on remaining portion (3.2% of leaves), d) leaf tip missing, grazed on remaining portion (14.2% of leaves).

Four possible situations were encountered for *S*. *alterniflora* leaves collected (Fig 2). To estimate area of missing tips, we took photos of leaves with tip missing and extended both edges of each leaf using ImageJ [26] until they intersected. We measured the taping angle (mean value ± standard error: 89.5° ± 0.2°). Then, assuming an isosceles triangle shaped-leaf, we calculated leaf area as tan(*Θ*)*(0.5*b*)^2^, where θ is the mean tapering angle and b is the basal width. From this we derived the width of the top edge at which the leaf was cut off and the tip missing (i.e., width of the isosceles triangle at the height of the leaf), using the relationship: *top width* = 2*Δh*/tan(*Θ*), where Δh is height difference between the reconstructed isosceles triangle and leaf height. In turn, the width of the top leaf edge was taken as the basal width for the isosceles triangle representing the missing tip, and the area of the missing tip was calculated from this width measurement and the mean tapering angle value. These estimates were included in the *S*. *alterniflora* leaf area allometric regression equation, Leaf Area_*calculated*_ = 0.3478 * Leaf Area_*measured*_ + 0.1383, (r^2^ = 0.91), to obtain final values of missing tip areas. The missing tip reconstruction approach was tested by severing 20 complete leaves at haphazardly-chosen cross sections along each leaf blade to determine an adjustment factor for our calculated missing tip areas: Missing Tip_*calculated*_ = 2.6498 * Missing Tip_*measured*_ + 0.2265, (r^2^ = 0.92).

Upon estimation of the area of the missing tip, the second step involved derivation of herbivory values assuming that 25%, 50%, or 75% of the missing tip had been consumed to illustrate a wide range of possible consumption scenarios. These calculations were done separately for each missing tip and the area of the bite marks added to the grazing estimation for the missing tip. Herbivory estimates were derived as percent leaf area consumed (cm^2^_grazed_ cm^-2^_leaf_), absolute leaf area consumed (cm^2^_grazed_ leaf^-1^), and absolute leaf mass consumed (grams dry weight leaf^-1^).

To assess leaf mass consumption, leaf area (cm^2^) to dry weight (g) conversion ratios were derived from additional subsamples: *S*. *alterniflora* (r^2^ = 0.88): Oven − Dry Weight = 0.0193 * Leaf Area − 0.1657; *A*. *germinans*: (r^2^ = 0.80): Oven-Dry Weight = 0.0197 * Leaf Area + 0.0252.

Cumulative leaf damage from grazers is a common proxy for herbivory [27, 28], but when compared between species, leaf lifespans should be taken into consideration. Leaves of *A*. *germinans* and *S*. *alterniflora* in the studied area have similar lifespans (100–150 days, [29, 30]); thus, our estimates should represent similar time scales for cumulative leaf damage.

#### Leaf nutrient content

Between 10–20 of the green *A*. *germians* or *S*. *alterniflora* leaves used to measure herbivory were oven dried (60°C to a constant weight) then ground into powder using a Thomas-Wiley plant tissue grinder and analyzed for percent carbon (%C) and nitrogen (%N) by mass using a CNS analyzer [31].

#### Leaf litter decomposition

The conventional litterbag method [23, 24] was used to estimate leaf decomposition rates. Nylon mesh bags (1.0 mm mesh) containing a known mass (air-dried) of green leaf detritus of *A*. *germinans* or *S*. *alterniflora* were fastened to rebar at the sediment surface within the plot. Leaves of both species begin to senesce while still attached to the plant, such that plucked senescent leaves would have been at different stages of decomposition. To normalize the stage of decomposition, we used green leaves for comparison. For measuring litterbag mass remaining, a conversion between oven- and air-dried material was necessary: *A*. *germinans* (r^2^ = 0.98): Air-dry weight = 1.8143 * Oven-Dry weight + 0.0477; *S*. *alterniflora* (r^2^ = 0.92): Air-dry weight = 1.3959 * Oven-Dry weight + 0.0186.

The following equation was fit for each vegetation type in each of the three stations:

$$\ln(\frac{{DM}_{retrievaL}}{{DM}_{deployment}})=C-k\Delta t$$

where DM_retrieval_ and DM_deployment_ correspond to the detritus mass (air-dry weight) remaining at retrieval and deployed at the beginning of the incubations, respectively; C is a constant, k is the decomposition rate, in days^-1^; and Δt is the time elapsed from deployment to retrieval, in days. The fits were done with least-square regression in SigmaPlot 12.3.

### Statistical analyses

We used the following model for the analysis of leaf nutrient content, herbivory, and decomposition:

(ResponseVariable)=Station+Subject+PlantType+Time+(PlantType*Time)+Error

where Station is a random blocking factor corresponding to each of the three locations where the plots were situated, Subject is a random factor, nested within Station, accounting for repeated measurements on individual organisms, Plant Type is a fixed factor corresponding to *A*. *germinans*, *S*. *alterniflora*-mixed or *S*. *alterniflora*-ref, and Time is a random, within-subject factor corresponding to collection date. For nutrient content, the response variable was percent nitrogen; for herbivory, the response variable was amount grazed (percent (%), absolute, or mass); and for decomposition, the response variable corresponded to ln(DM_retrieval_DM_deployment_). All values obtained from a given plot on each sampling date were averaged into one true replicate. We used R version 3.2.4 [32] with package “lme4” [33] for analysis. A likelihood ratio test was used to determine significance of each factor: (1) two models were constructed for each comparison, the full model and a model lacking the factor of interest; (2) a likelihood ratio test using the “anova ()” function provided a comparison between the models; and (3) any differences between the models are attributed to the factor of interest [34]. When comparing models for a fixed factor, maximum likelihood estimators were used in the full and partial models; for random factors, restricted maximum likelihood estimators were used for both models being compared [34, 35]. Post-hoc comparisons were computed for specific factor levels following the same approach. Recent, robust statistical analysis developments have allowed for inclusion of several factors to be accounted for smaller sample sizes (e.g., n = 3), though the statistical power may still be reduced [33–35].

## Results

### Leaf herbivory

**Fig 3:**
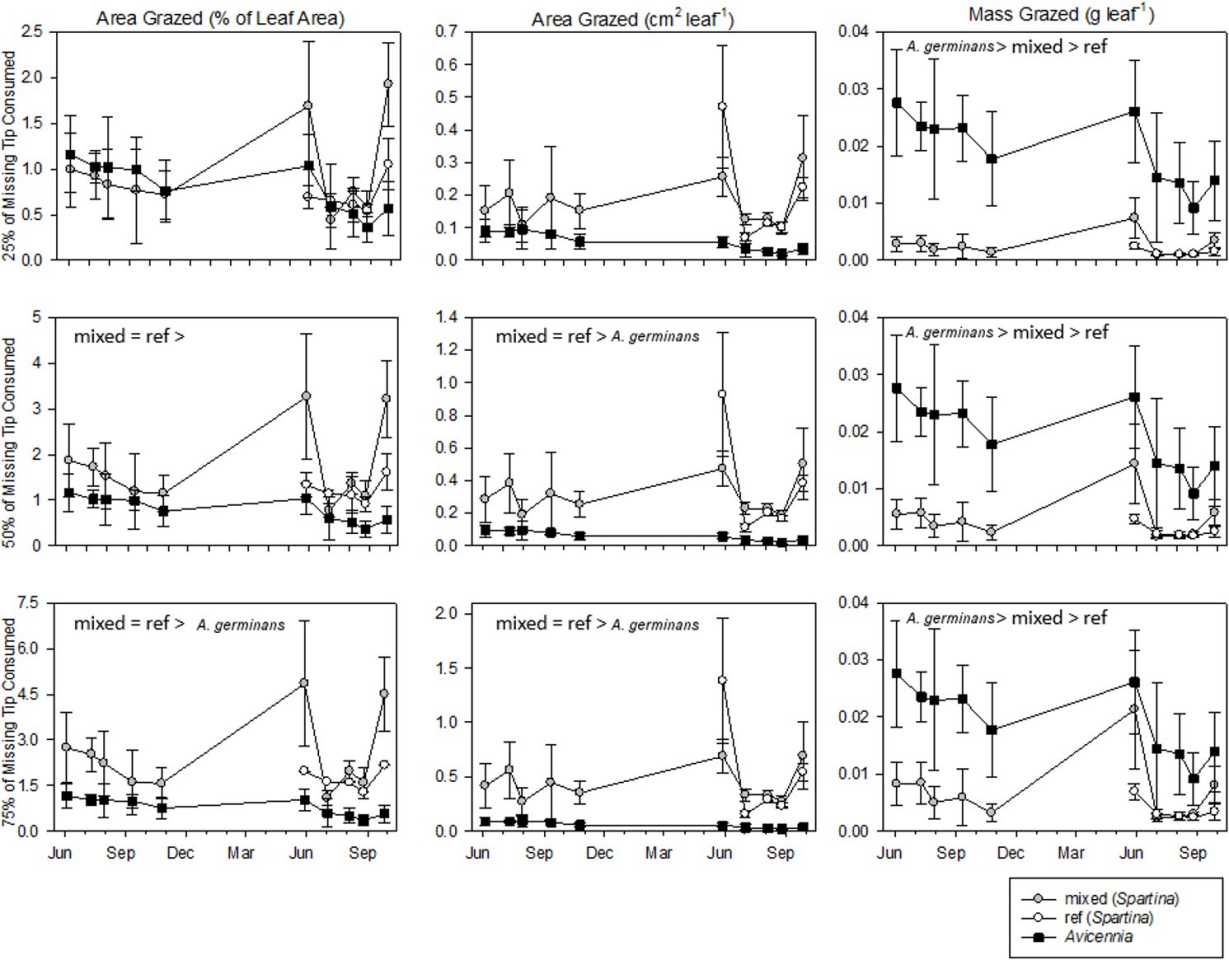
Leaf herbivory of *A*. *germinans* and *S*. *alterniflora*-mix (6 separate analyses) over 2012–2013. Grazing on leaf tissue was compared on the bases of percentage of total leaf area (left column plots), absolute leaf area (center column), and absolute leaf mass (right column). Note y-axes’ scales vary in left and center columns. Values between top, middle, and bottom rows vary only for *S*. *alterniflora*, with different assumptions of missing tip consumption (top: 25%, middle: 50%, bottom: 75%).

Herbivores removed more leaf area of *S*. *alterniflora* but consumed more leaf mass of *A*. *germinans*, per leaf. Fig 3 shows consumption (*S*. *alterniflora*: portion of overall leaf removal; *A*. *germinans*: equal to leaf removal), but overall leaf removal would account for 100% of missing tips (Yearly Leaf Removal Averages; Percent Area: *S*. *alterniflora*-mixed: 3.66%, *S*. *alterniflora*-ref: 2.22%, *A*. *germinans*: 0.75% and Absolute Leaf Area: *S*. *alterniflora*-mixed: 0.60 cm^2^ leaf^-1^, *S*. *alterniflora*-ref: 0.68 cm^2^ leaf^-1^, *A*. *germinans*: 0.06cm^2^ leaf^-1^). Significantly more area was removed from each *S*. *alterniflora* leaf than each *A*. *germinans* leaf. On a mass basis, even if 75% of missing *S*. *alterniflora* tips were consumed, more mass per leaf would be entering herbivores via *A*. *germinans* than via *S*. *alterniflora* (Fig 3 and Table 1). Post-hoc analysis did not reveal any significant differences in herbivory between *S*. *alterniflora*-mixed and *S*. *alterniflora*-ref. *A*. *germinans* leaves were more frequently damaged (63.1% of leaves intact; 36.9% of leaves grazed) than *S*. *alterniflora* leaves (See Fig 2; 76.1% intact, no grazing; 6.4% intact, grazed; 3.2% not intact, no grazing; 14.2% not intact, grazed).

**Table 1:** Chi-squared (χ^2^), degrees of freedom (df), and p-values of factors within Likelihood Ratio Test: Station, Plant Type (*A*. *germinans*, *S*. *alterniflora*-mixed, *S*. *alterniflora*-ref), Date, and interaction of Plant Type x Date. Bold P values indicate significance (p < 0.05).

Response Variable	Station		Plant Type		Date		Plant Type x Date	
	χ^2^ (df)	p-value	χ^2^ (df)	p-value	χ^2^ (df)	p-value	χ^2^ (df)	p-value
**Nutrients** **(2013)**								
% Leaf Nitrogen	0 (1)	>0.999	15.5 (2)	**<0.001**	15.7 (1)	**<0.001**	0 (1)	>0.999
**Herbivory: *A*. *germinans*/*S*. *alterniflora*-mixed** **(2012 and 2013)**								
*Percent Leaf Area Consumed*								
25%	0 (1)	>0.999	0.7 (1)	0.397	0 (1)	>0.999	1.0 (1)	0.325
50%	0 (1)	>0.999	8.4 (1)	**0.004**	0 (1)	>0.999	1.1 (1)	0.292
75%	0 (1)	>0.999	12.4 (1)	**0.001**	0 (1)	>0.999	1.2 (1)	0.275
*Absolute Leaf Area Consumed*								
25%	0.1 (1)	0.807	9.5 (1)	**0.002**	0 (1)	>0.999	0 (1)	>0.999
50%	0 (1)	0.874	12.3 (1)	**<0.001**	0 (1)	>0.999	0 (1)	>0.999
75%	0 (1)	0.909	13.3 (1)	**<0.001**	0 (1)	>0.999	0 (1)	>0.999
*Absolute Leaf Mass Consumed*								
25%	0 (1)	>0.999	16.7 (1)	**<0.001**	0 (1)	>0.999	0.5 (1)	0.466
50%	0 (1)	>0.999	14.5 (1)	**<0.001**	0 (1)	>0.999	0.7 (1)	0.417
75%	0 (1)	0.999	11.6 (1)	**<0.001**	0 (1)	>0.999	0.9 (1)	0.356
**Herbivory: *A*. *germinans*/*S*. *alterniflora*-mixed/*S*. *alterniflora*-ref** **(2013)**								
*Percent Leaf Area Consumed*								
25%	0 (1)	>0.999	2.4 (2)	0.294	0 (1)	>0.999	2.0 (1)	0.157
50%	0 (1)	0.917	6.5 (2)	**0.038**	0 (1)	>0.999	2.1 (1)	0.144
75%	0.1 (1)	0.717	8.6 (2)	**0.014**	0 (1)	0.950	2.0 (1)	0.159
*Absolute Leaf Area Consumed*								
25%	0 (1)	>0.999	5.2 (2)	0.075	0.2 (1)	0.640	3.3 (1)	0.068
50%	0 (1)	>0.999	6.7 (2)	**0.035**	0.5 (1)	0.485	3.0 (1)	0.082
75%	0 (1)	>0.999	7.2 (2)	**0.028**	0.6 (1)	0.442	2.9 (1)	0.086
*Absolute Leaf Mass Consumed*								
25%	0 (1)	>0.999	19.9 (2)	**<0.001**	0 (1)	>0.999	0 (1)	>0.999
50%	0 (1)	>0.999	15.3 (2)	**<0.001**	0 (1)	>0.999	0 (1)	0.989
75%	0 (1)	>0.999	10.7 (2)	**0.005**	0 (1)	>0.999	0.2 (1)	0.656
**Decomposition** **(2012)**								
*Percent Mass Remaining*								
	0 (1)	>0.999	3.2 (1)	0.075	19.5 (1)	**<0.001**	27.8 (3)	**<0.001**

### Leaf nutrient content

**Fig 4:**
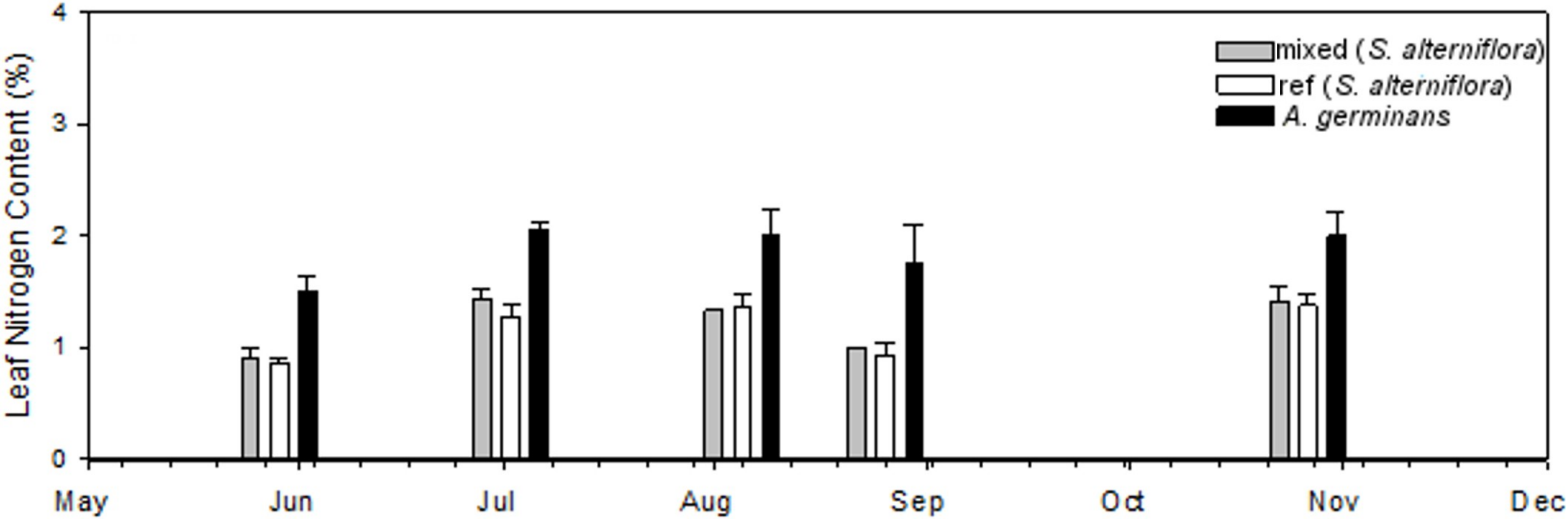
Percent nitrogen (%N) leaf content in 2013 for all treatments. *S*. *alterniflora*-mixed %N did not vary significantly from *S*. *alterniflora*-ref %N (p = 0.055), and *A*. *germinans* %N was greater than *S*. *alterniflora*-mixed %N (p < 0.004) and *S*. *alterniflora*-ref (p < 0.002).

Leaf nitrogen content was higher in *A*. *germinans* leaves than in *S*. *alterniflora* leaves (p < 0.05, Fig 4 and Table 1), with %N in *S*. *alterniflora* leaves not varying significantly between *S*. *alteriflora*-mixed and *S*. *alterniflora*-ref. These differences were consistent over time (Plant Type x Time: p > 0.05, Table 1).

### Leaf decomposition

**Fig 5:**
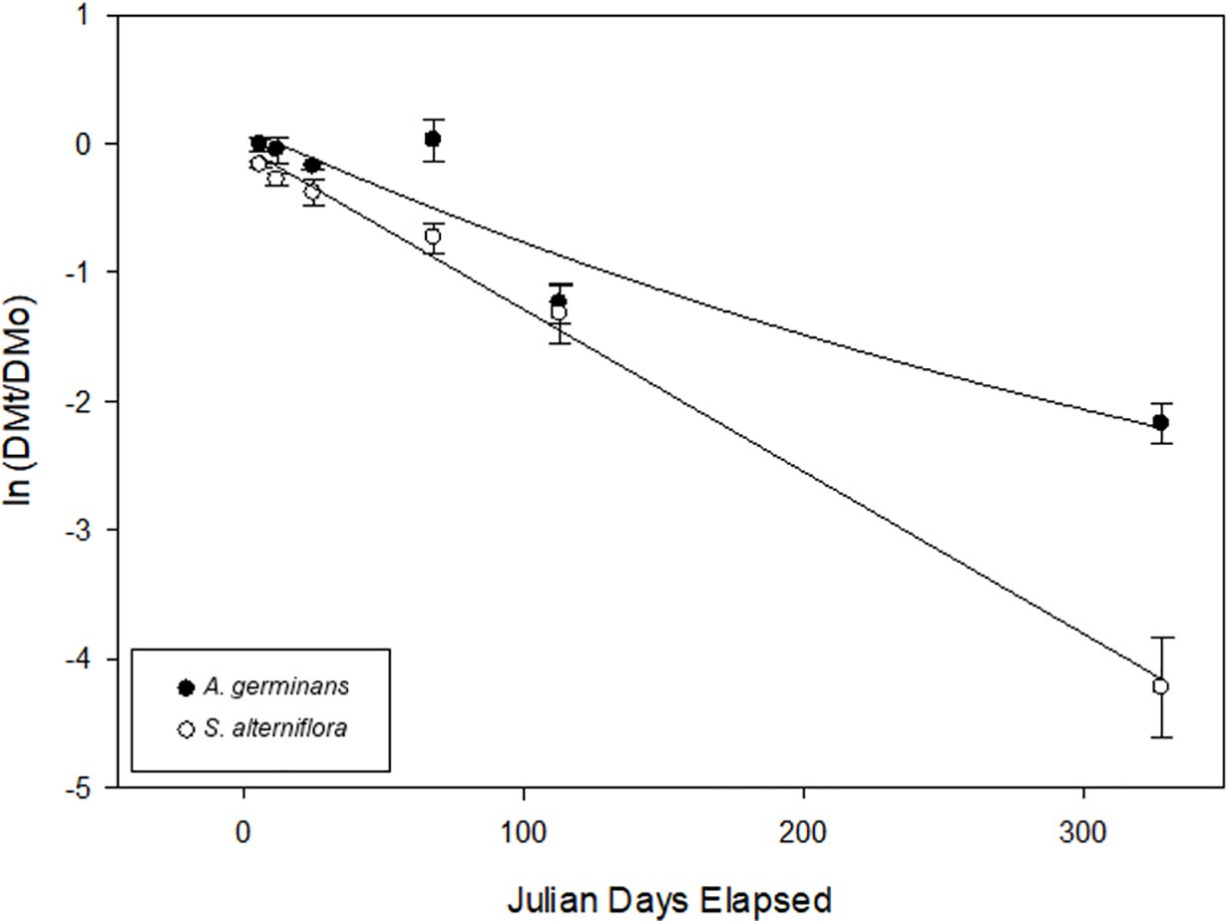
Decomposition of freshly senesced *A*. *germinans* and *S*. *alterniflora* leaf detritus. Y values, ln(DMt/DMo), are the natural logarithms of the proportion of detrital mass remaining at time, t. Duplicate bags of each plant type were collected 6, 12, 25, 68, 113, and 328 days after deployment. Upon retrieval, the samples were rinsed off with fresh water, and the contents within the bag carefully removed and sieved through a 0.5mm mesh. Samples were oven-dried (60°C to constant mass) and converted to an air-dried weight using regressions obtained with additional samples. Least square regression for single exponential models (p < 0.05, *A*. *germinans*: r^2^ = 0.90; *S*. *alterniflora* r^2^ = 0.95). *A*. *germinans* leaf detritus decomposed slower than *S*. *alterniflora* leaf detritus (p < 0.05, Table 1).

Leaf decomposition was faster for *S*. *alterniflora* than for *A*. *germinans* leaves (Plant Type x Time: p < 0.001, Fig 5 and Table 1). Similar leaf detritus weights for the two plant types remained in the bags over the first collection times, but 328 days after deployment only ca. 2% of the initial weight remained for *S*. *alterniflora* as opposed to ca. 15% for *A*. *germinans* leaf detritus. We used the single exponential decay model because other models (such as the linear or double exponential) did not improve the quality of the fit (S1 and S2 Figs).

## Discussion

There was a greater mass consumed from *A*. *germinans* leaves than *S*. *alterniflora* leaves, even when assuming 75% of each missing *S*. *alterniflora* tip was consumed (*A*. *germinans*: 0.018 g leaf^-1^, *S*. *alterniflora*-mixed: 0.007 g leaf^-1^, *S*. *alterniflora*-ref: 0.004 g leaf^-1^, Fig 3 and Table 1), and these differences were consistent over time (Plant Type x Time: p > 0.35). Based on previous degrees of herbivory in other systems [15], 0% and 75% consumptions are unlikely; we chose to include analysis of a conservative 75% scenario to highlight the consumption relative to *A*. *germinans*. In addition to the greater mass consumption per leaf, mangrove leaves contained 48–63% greater nitrogen concentrations than *S*. *alterniflora* leaves (*A*. *germinans*: 1.86%, *S*. *alterniflora*-mixed: 1.25%, *S*. *alterniflora*-ref: 1.14%, Fig 4 and Table 1), suggesting herbivores may be targeting *A*. *germinans* for their higher nutritional value. Typical leaf nitrogen content ranges from 1–4% [36], indicating *S*. *alterniflora* is relatively nitrogen poor. Onuf et al. [37] fertilized red mangroves to the extent of increasing leaf nitrogen concentrations by 33%, resulting in a 400% increase in herbivory. The leaf nitrogen concentration discrepancy of 48–63% observed in our study may influenced grazer preference of leaves.

Other factors such as lignin concentration, tannins, and silica content [38–40] can affect palatability. Relative to other mangrove genuses, *A*. *germinans* leaves contain fewer tannins (0.8% DW, [41]) but still more than *S*. *alterniflora* (0.4% DW, [42]). The most likely dominant grazers of *A*. *germinans* in our system are grasshoppers, based upon identification of nearby marsh grazing populations [43]. The most likely dominant grazer of *S*. *alterniflora* is the marsh periwinkle *Littoraria irrorate* [44], who incidentally consumes *S*. *alterniflora* as it consumes fungus that infects *S*. *alterniflora* [45]. The results demonstrate *A*. *germinans* may be a preferable option to certain grazers in the nGOM. Despite this apparent grazer preference of live leaf tissue, the morphology differences between *A*. *germinans* and *S*. *alterniflora* leaves lend to a greater impact on *S*. *alterniflora*, the less consumed plant.

Primary production is a function of leaf area, and leafy plants have exhibited several adaptations to exposure of leaf surface area to sunlight [46, 47]. Grazing incidentally decreases leaf area, but to sustain the same level of secondary production, more leaf area of one species of plant may need to be consumed (or removed through sloppy feeding, wherein material is removed from the plant but not consumed by the grazer) than the leaf area of another plant species. There is a greater mass per unit area in *A*. *germinans* leaves than in *S*. *alterniflora* leaves, but the overall effect of herbivory on either plant species is small. If herbivores are 100% responsible for the missing areas, they are still only removing <5% of leaf area in cordgrass and <1% of leaf area in mangroves, but this does not account for entire leaves that are removed from the plants. No published studies quantifying herbivory on *A*. *germinans* along the nGOM could be found, but in warmer climates, higher rates of herbivory were observed on *A*. *germinans* (0.83–4.5% of leaf area in Guadeloupe [28]; 8–36% in Belize [27]). Alongi [48] asserts that usually <10% of mangrove defoliation is attributed to insect herbivory. As such, most leaf biomass ends up as detrital matter.

Carbon, nitrogen, and energy also make their way into the food chain via detrital pathways: detritivores and decomposers extract a portion of this sustenance before it is either exported out of the system or sequestered in the wetland peat. Higher leaf nutrient content may result in faster decomposition [49–51], yet we found slower decomposition of the nitrogen-rich mangrove leaves than the nitrogen-poor cordgrass leaves. This counter-intuitive result could be influenced by potentially stronger factors, such as higher lignin concentrations or waxier cuticle [50, 52], tannins, alternative nutrient limitation (i.e., phosphorus), or different microbial communities [19]. While marine systems are often nitrogen-limited, Johnson et al. [53] documented phosphorus limitation in seagrass beds nearby, suggesting detritivores may have preferred phosphorus-rich material. Phosphorus levels in leaves may have varied significantly but were not measured. Studies in this region indicate leaf phosphorus concentrations are similar between *A*. *germinans* (0.11–0.15%, [54]) and *S*. *alterniflora* (0.08–0.15%, [55]). Perry and Mendelssohn [56] found *A*. *germinans* leaves decomposed faster than *S*. *alterniflora* leaves in an area with a long-established population of *A*. *germinans*, though microbial communities can vary substantially with relative mangrove influence [57]. Microclimate conditions between litterbags of each study were unlikely to have a significantly different effect, despite the variable dimensions and mesh sizes [58].

In this study, we found contrasting perspectives by investigation of first order consumption. Between *A*. *germinans* and *S*. *alterniflora* leaves, chewing herbivores consumed more *A*. *germinans* leaf material while causing less damage to the leaf, perhaps enticed by the higher nitrogen content. Chewing herbivores removed more leaf area from *S*. *alterniflora* but ingested less plant material. *S*. *alterniflora* leaves’ greater area to mass ratio likely also contributed to its more rapid decomposition. The introduction of new producers like *A*. *germinans* within a system presents herbivores (and detritivores) another food choice. The magnitude of subsequent shifts in trophic processes will align with the extent of the new producer’s dominance within the native system; however, those magnitudes will also align with the relative preference of first order consumers to a producer growing in that location, which may differ from that producer’s growth in its native environment. By understanding dichotomies of herbivory and decomposition at range extremes, we can better identify and explain the impending changes to shifts associated with economically and ecologically valuable systems, like *S*. *alterniflora*-dominated salt marsh ecosystems of the nGOM. We look forward to future research identifying changes in trophic processes with mangrove expansion and/or changes in the marsh communities alongside other climate-motivated species range expansions.

## Supporting information

S1 Fig Linear decomposition model. Y-axis: dry mass at time t divided by initial dry mass. *A*. *germians*: r^2^ = 0.89; *S*. *alterniflora*: r^2^ = 0.95.
(TIF)

S2 Fig Double-exponential decomposition model. Y-axis: dry mass at time t divided by initial dry mass. *A*. *germinans*: r^2^ = 0.90; *S*. *alterniflora*: r^2^ = 0.99.
(TIF)
