# Supplementary figures and images for: Tropicalization of the barrier islands of the northern Gulf of Mexico: A comparison of herbivory and decomposition rates between smooth cordgrass (Spartina alterniflora) and black mangrove (Avicennia germinans)
Source: PLoS One. 2019 Jan 7;14(1):e0210144. doi: 10.1371/journal.pone.0210144 (PMC6322730; doi:10.1371/journal.pone.0210144)

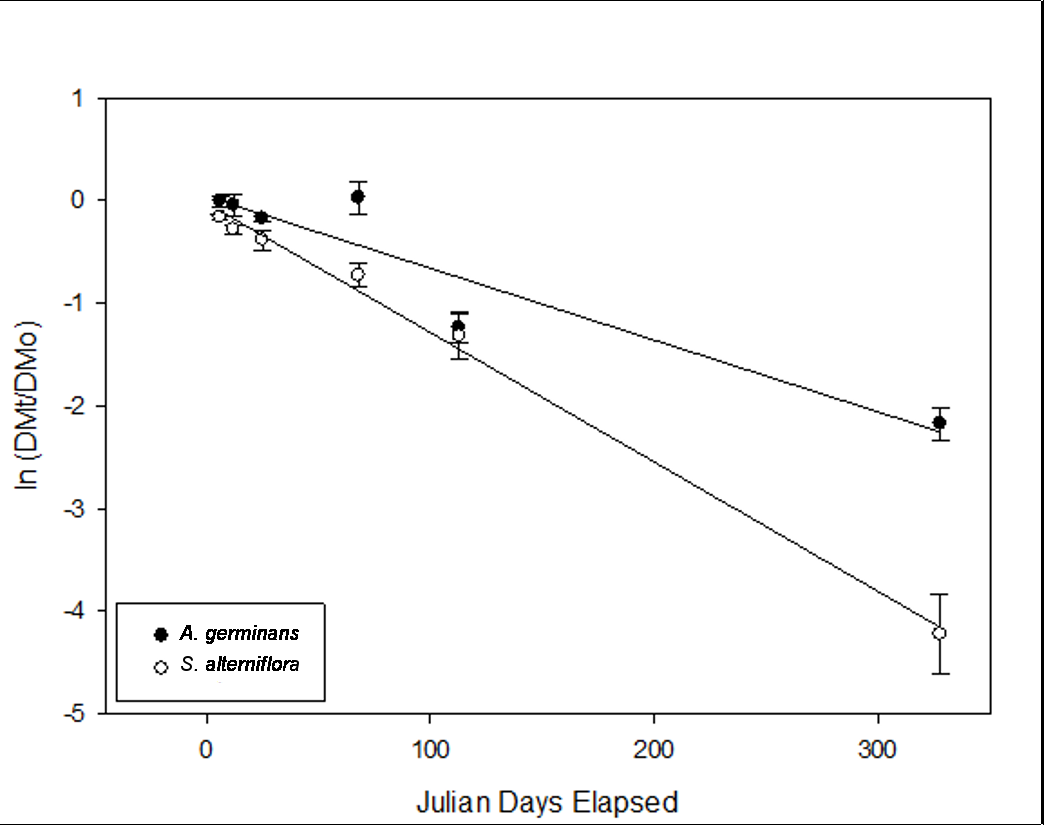

Supplement: S1 Fig — Y-axis: dry mass at time t divided by initial dry mass. A. germians: r2 = 0.89; S. alterniflora: r2 = 0.95. (TIF) [file pone.0210144.s001.tif]

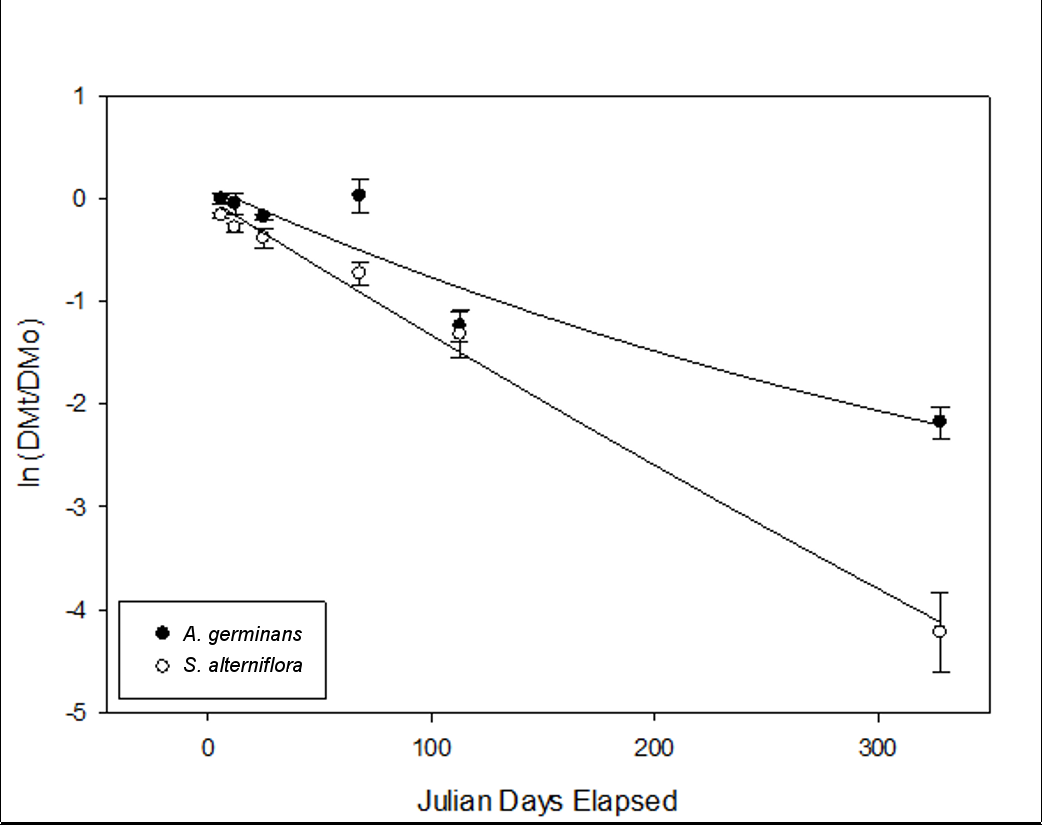

Supplement: S2 Fig — Y-axis: dry mass at time t divided by initial dry mass. A. germinans: r2 = 0.90; S. alterniflora: r2 = 0.99. (TIF) [file pone.0210144.s002.tif]
